# Effects of species and geo-information on the ^137^Cs concentrations in edible wild mushrooms and plants collected by residents after the Fukushima nuclear accident

**DOI:** 10.1038/s41598-021-01816-z

**Published:** 2021-11-17

**Authors:** Masabumi Komatsu, Shoji Hashimoto, Toshiya Matsuura

**Affiliations:** 1 Department of Mushroom Science and Forest Microbiology, Forestry and Forest Products Research Institute, Tsukuba, Ibaraki 305-8687 Japan; 2 Department of Forest Soils, Forestry and Forest Products Research Institute, Tsukuba, Ibaraki 305-8687 Japan; 3 Tohoku Research Center, Forestry and Forest Products Research Institute, Morioka, Iwate 020-0123 Japan

**Keywords:** Environmental impact, Forest ecology, Pollution remediation, Ecological modelling

## Abstract

After the accident at the Fukushima Daiichi Nuclear Power Plant (FDNPP), much of the wild and edible mushrooms and plants in the surrounding areas were contaminated with radiocesium (^137^Cs). To elucidate their concentration characteristics, we analyzed ^137^Cs radioactivity data in edible forest products brought in for food inspection by the residents of Kawauchi Village, 12–30 km away from the FDNPP, from 2012 to 2019. A Bayesian model to estimate ^137^Cs concentration was constructed. Parameters of the normalized concentration of species (*NC*_sp_) for mushrooms were similar to those of the same species obtained in a previous study. Although *NC*_sp_ values were highly varied among species, mycorrhizal mushrooms tended to have high *NC*_sp_ values, followed by saprotrophic mushrooms, and wild edible plants values were low. Also, half of mycorrhizal mushroom species (8 of 16) showed an increasing trend in concentration with time; however, saprotrophic mushrooms and wild plants generally demonstrated a decreasing trend (22 of 24). The model considering the sub-village location information decreased the error of individual samples by 40% compared to the model not considering any location information, indicating that the detailed geo-information improved estimation accuracy. Our results indicate that the radioactivity data from samples collected by local residents can be used to accurately assess internal exposure to radiation due to self-consumption of contaminated wild mushrooms and plants.

## Introduction

In March 2011, the Fukushima Daichi Nuclear Power Plant (FDNPP) accident triggered by the big earthquake and tsunami induced extensive contamination with radioactive nuclides in eastern Japan lands. Because of the explosion, several types of radioactive nuclides were released into the atmosphere. The released amounts of ^133^Xe, ^131^I and ^137^Cs were estimated as 6000–12,000 PBq^1^, 100–500 PBq^2^ and 15–20 PBq^3^, respectively. Moreover, almost the same amount of ^134^Cs was released as ^137^Cs^3^. Since about 70% of Japan’s lands are covered with forests^4^, the forest became the most widely contaminated area with radioactive nuclides. Among radioactive nuclides released from the FDNPP, radiocesium (^134^Cs and ^137^Cs) is the most important contamination source due to its high emission amounts and persistence in terrestrial environments. Especially, prolonged contamination by ^137^Cs, which has longer physical half-lives (30.17 years) than ^134^Cs (2.065 years), is concerned. The total amount of ^137^Cs deposited on land because of the accident is estimated to be 2–6 PBq^3^. Within a few years of the accident, most of the radiocesium deposited on forests transferred to the surface layer of mineral soils (81–88% in 6 of 9 sites in 2015)^5^. The outflow of radiocesium from the forest is small (annually less than 0.3% of total deposition)^6,7^, and model analysis demonstrated that the ^137^Cs flux between tree an soil would converge to ~ 1% of total deposition in both directions^8^. Therefore, some amount of radiocesium is expected to be circulated in the forest ecosystem.

Radioactive materials cause exposure to humans through several ways such as external exposure by radiation in the environment and internal exposure via foods. In particular, the risk of internal exposure because of self-ingestion containing high level of radiocesium has attracted considerable attention from researchers, thereby necessitating some means to reassure the public regarding safety. Since April 2012, the Japanese government set the criteria of radioactive cesium (sum of ^134^Cs and ^137^Cs) concentration in general foods as 100 Bq/kg to make annual internal exposure by food ingestion to < 1 mSv per year^9^. In municipalities where food products are reported to exceed the standard criteria, shipping restrictions will be imposed. Ten years have passed after the accident, and the contamination of agricultural products in the affected areas has gradually decreased. For example, the shipping of tuberous leafy vegetables such as cabbage was restricted at 58 municipalities in Fukushima Prefecture in March 2011; however, as of 2021, the restrictions have been lifted except for seven municipalities around the FDNPP^10^. However, the shipping of wild forest foods such as mushrooms, and edible plants is still extensively limited. The numbers of restricted municipalities for shipment are 117 for wild mushrooms and 113 for koshiabura (*Eleutherococcus* (*Chengiopanax*) *sciadophylloides*), the most extensively restricted species among wild plants as of 2021. Especially for wild mushrooms, there are many species, and the concentration characteristics of each species are not clear; the shipping restrictions are set uniformly without distinction of species.

Wild mushrooms and plants are favored in the mountainous village areas of Japan; they are deeply involved in the local culture through a leisure activity of gathering and providing the collected products^11^. A questionnaire survey conducted to residents of Kawauchi Village revealed that ~ 70% of households used to enjoy gathering wild mushrooms and plants before the accident; however, after the accident, the ratio decreased to ~ 20%^12^. The radiocesium contamination has deeply affected local food culture and leisure activities in mountainous communities. Furthermore, even in municipalities where shipments were restricted, the private consumption of wild mushrooms and wild plants itself was not restricted (except for three municipalities of wild mushrooms^10^). There are concerns of excess internal exposure because of the self-consumption of wild mushrooms and plants. Since the preferable wild mushrooms and plants are regionally variant, it is essential to know each species concentration characteristics. After the Chernobyl accident in 1986, the persistence of forest foods contamination has still been reported in Europe^13,14^, and it is also necessary to show the temporal trends of these concentrations for prospects.

In Fukushima and neighboring prefectures after the FDNPP accident, monitoring of radioactivity in wild mushrooms^15–18^ and wild plants^19–21^ has been reported. However, most of the monitoring has been done at fixed points, and the characteristics of radionuclide concentration associated with a species has not been verified across regions. Moreover, the role of regional bias in concentration data has not been sufficiently studied. Komatsu et al.^22^ analyzed the public extensive radioactivity data of wild mushrooms collected for the food monitoring by local governments. They analyzed using mushroom data from 3019 samples of 107 species collected in 265 municipalities by the model. In the model, the radiocesium concentration in wild mushrooms normalized by the radiocesium deposition was simultaneously analyzed for the effects of species and collected municipalities. Because there were many measurement results below the detection limit, the model was designed to evaluate these values using censored distribution. Furthermore, a spatial autocorrelation was assumed in the effect of the sampling municipality. In this manner, the bias because of collection sites and species bias were avoided as much as possible. The parameter of normalized concentration of species (*NC*_sp_) was evaluated as a characteristic of radiocesium concentration in species; it was reported that the concentration characteristics varied more than 100 folds (up to 209 times) among wild mushroom species. The effects of species and regions on radiocesium concentrations are useful information when considering shipping restrictions and estimating internal exposure due to ingestion. However, it was necessary to confirm the universality of the concentration characteristics using different data sets. Additionally, location information is based on municipalities that occupy areas ranging from dozens to hundreds of square kilometers, and such areas display a variable range of radiocesium deposition. Thus, it has been difficult to precisely describe the effect of region on the radioactivity of wild foods.

**Figure 1:**
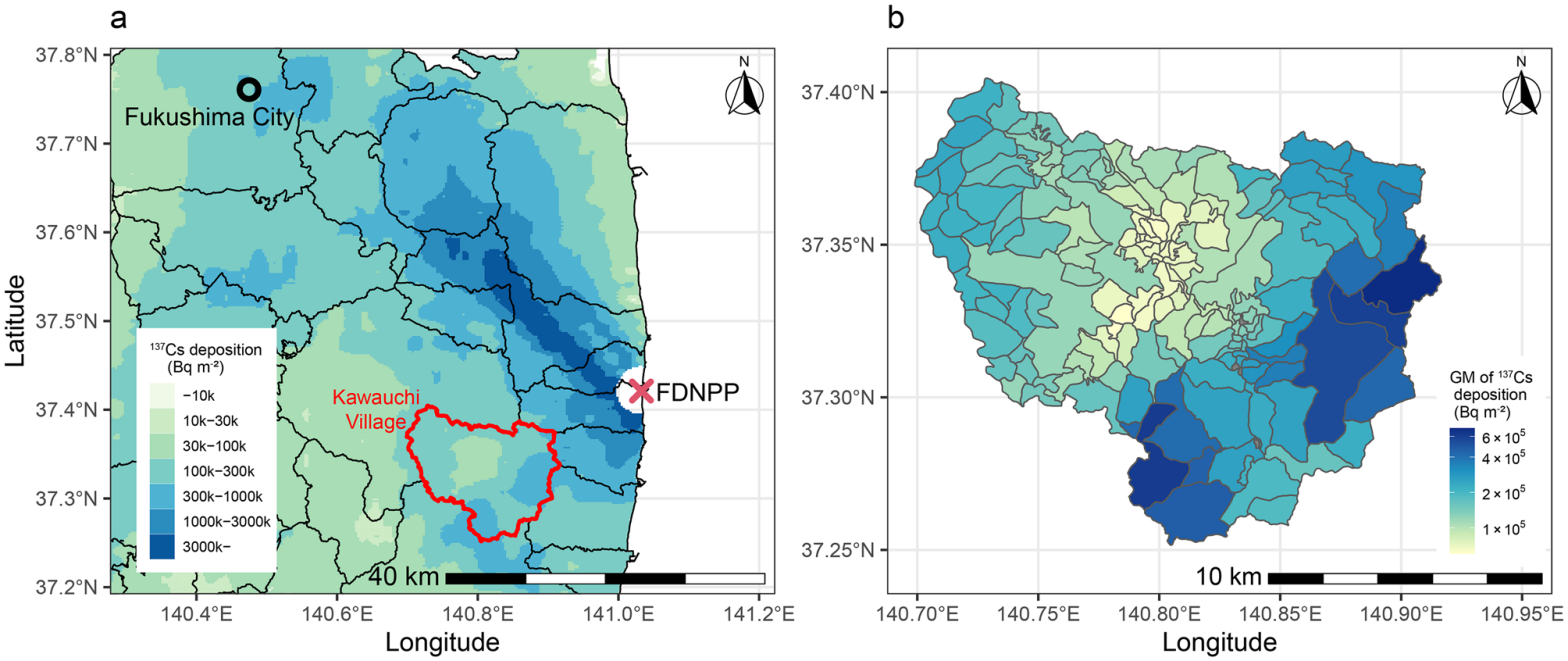
(**a**) Location of Kawauchi Village. ^137^Cs deposition amount provided by Kato and Onda^26^ is filled on the map. The municipal boundaries were generated from Administrative Zones Data^27^. (**b**) The sub-village (“Ko-aza”) boundaries of Kawauchi Village. The geometric mean (GM) of ^137^Cs deposition by Kato and Onda^26^ was shown for each sub-village. The sub-village boundaries were created from a hand-drawn map obtained from the administrative office. The ^137^Cs deposition data in the plotted areas were based on the results of the third airborne monitoring survey performed by the Ministry of Education, Culture, Sports, Science and Technology (MEXT) from May 31 to 2 July 2011^28^. These maps were created using R version 4.1.0^29^ and ggplot2 package^30^.

Therefore, we focused on the data of the inspection of foods for self-consumption conducted in Kawauchi Village, Fukushima Prefecture (Fig. 1). After the FDNPP accident, Fukushima and surrounding prefectures set up inspection facilities with measurement systems in their municipalities for screening foods brought by residents. The inspection results in some municipalities are analyzed to evaluate the internal dose of local residents^23–25^. In Kawauchi Village, the radioactivity of multiple wild mushroom and plants have been inspected since 2012 to the present. Information on collection areas of sub-village levels, called “Ko-aza” in Japanese, is recorded. Therefore, we considered that the data of the brought in inspection in Kawauchi Village would possess high value as data for inter-species and inter-region analysis on the wild mushrooms and edible plants’ radioactivity concentrations.

In this study, we analyzed the inspection results of wild mushrooms and plants brought in by residents in Kawauchi Village, Fukushima Prefecture and examined the effects of species, temporal trends, and collection areas on the radiocesium concentrations. The results of wild mushrooms were compared with the results of Komatsu et al.^22^, who analyzed data from a wide area in eastern Japan to confirm the universality of the concentration characteristics. Furthermore, we aimed to quantify the concentration characteristics of edible wild plants, which have not been shown to date, and to show the time-dependent trends of radiocesium concentrations of mushrooms and wild plants. Finally, by analyzing the data with detailed location information, we confirmed whether the estimation accuracy could be improved.

## Results

### Estimation results of the two models

From the food inspection results in Kawauchi Village, ^137^Cs concentration data of wild mushrooms and plants for a total of 4534 specimens of 40 species taken from May 2012 to March 2020 were obtained (Table ﻿1). Among these data, 1834 specimens had measured values below the detection limit. Tables S1 and S2 show the data of specimens and sub-village, respectively. In the analysis, two models (“sub-village model” and “whole village model”) were used. Both models considered the species, date of collection, and ^137^Cs deposition amount as explanatory variables. *NC*_sp_ and *λ*_sp_ are the concentration parameters of species standardized by deposition and slope parameter with time, respectively. Furthermore, the sub-village model hypothesized that each sub-village shown in Fig. 1b would have effects on sample ^137^Cs concentration (defined as *r*_loc_) dependent to the deposition (*D*_loc_). The whole village model did not consider sample location in the village and used geometric mean of ^137^Cs deposition for whole village (*D*_village_) in the analysis.

**Table 1:** Posterior distribution of normalized concentration (*NC*_sp_), temporal change parameter (*λ*_sp_), and effective annual change ratio (*R*_eff_sp_) of each species.

Group	Subgroup	Species	Common name, edible part	N (detected)^4)^	log_10_ *NC*_sp_ (m^2^ kg^−1^, FM)					*λ*_sp_ (year^−1^)					*R*_eff_sp_ (%)			Half life or doubling time (year)^5)^
					Median	Mean	sd	CI2.5%	CI 97.5%	Median	Mean	sd	CI2.5%	CI 97.5%	Median	CI2.5%	CI 97.5%	
Mushroom	Mycorrhizal	*Albatrellus confluens*	Ningyotake (ja)^3)^	79 (69)	− 3.59	− 3.59	0.08	− 3.74	− 3.44	− 0.005	− 0.004	0.030	− 0.062	0.054	97	85	111	− 20.7
		*Astraeus hygrometricus*	Hygroscopic earthstar	48 (26)	− 3.95	− 3.95	0.08	− 4.12	− 3.78	− 0.047	− 0.048	0.039	− 0.127	0.026	88	73	104	− 5.3
		*Boletopsis grisea*	Kurokawa (ja)	62 (62)	− 2.58	− 2.58	0.07	− 2.71	− 2.44	0.041	0.042	0.025	− 0.006	0.091	107	96	121	9.6
		*Cortinarius caperatus*	Gypsy mushroom	11 (11)	− 1.68	− 1.68	0.16	− 1.99	− 1.36	− 0.009	− 0.009	0.047	− 0.104	0.085	96	77	119	− 15.9
		*Cortinarius salor, C. iodes*	Murasakiaburashimeji (ja) or Spotted cort	19 (19)	− 1.68	− 1.67	0.13	− 1.93	− 1.42	0.011	0.011	0.047	− 0.080	0.107	100	81	125	242.3
		*Cortinarius tenuipes*	Kurifusentake (ja)	21 (21)	− 2.87	− 2.87	0.11	− 3.09	− 2.64	0.001	0.002	0.040	− 0.075	0.082	98	82	118	− 32.9
		*Entoloma sarcopum*	Urabenihoteishimeji (ja)	15 (13)	− 3.11	− 3.10	0.14	− 3.37	− 2.84	0.052	0.054	0.047	− 0.035	0.153	110	90	139	7.2
		*Hygrophorus russula*	Pinkmottle woodwax	67 (67)	− 2.08	− 2.08	0.07	− 2.21	− 1.96	0.019	0.019	0.031	− 0.042	0.080	102	89	118	35.2
		*Lactarius volemus*	Weeping milk cap	26 (25)	− 2.58	− 2.57	0.10	− 2.78	− 2.37	0.000	0.001	0.042	− 0.077	0.084	98	82	119	− 31.3
		*Lyophyllum fumosum*	Shakashimeji (ja)	55 (51)	− 3.36	− 3.36	0.08	− 3.52	− 3.20	0.026	0.028	0.031	− 0.032	0.089	104	91	120	18.5
		*Lyophyllum shimeji*	Honshimeji (ja)	30 (28)	− 3.32	− 3.32	0.12	− 3.56	− 3.07	0.041	0.043	0.044	− 0.040	0.132	107	89	132	9.7
		*Ramaria botrytis*	Clustered coral	23 (23)	− 2.44	− 2.44	0.11	− 2.65	− 2.23	0.004	0.004	0.036	− 0.068	0.076	99	84	116	− 51.9
		*Sarcodon aspratus*	Kotake (ja)	531 (530)	− 2.36	− 2.36	0.03	− 2.42	− 2.31	0.094	0.094	0.011	0.073	0.115	121	116	127	3.6
		*Suillus bovinus*	Jersey cow mushroom	90 (88)	− 2.75	− 2.75	0.06	− 2.87	− 2.63	− 0.012	− 0.012	0.030	− 0.071	0.047	95	83	109	− 14.0
		*Tricholoma equestre*	Yellow knight mushroom	18 (17)	− 2.95	− 2.95	0.12	− 3.19	− 2.70	− 0.049	− 0.049	0.050	− 0.148	0.045	87	69	108	− 5.1
		*Tricholoma matsutake*	Matsutake (ja)	53 (53)	− 2.80	− 2.79	0.08	− 2.94	− 2.65	0.027	0.027	0.027	− 0.027	0.080	104	92	118	17.7
	Saprotrophic	*Armillaria mellea*	Honey mushroom	71 (57)	− 3.62	− 3.62	0.07	− 3.75	− 3.49	− 0.038	− 0.038	0.032	− 0.104	0.024	90	77	103	− 6.3
		*Armillaria tabescens*	Ringless honey mushroom	21 (13)	− 3.86	− 3.86	0.12	− 4.11	− 3.63	− 0.047	− 0.046	0.043	− 0.135	0.040	88	72	107	− 5.3
		*Grifola frondosa*	Hen of the woods	60 (35)	− 3.97	− 3.97	0.08	− 4.13	− 3.82	− 0.034	− 0.034	0.029	− 0.090	0.022	90	79	103	− 6.8
		*Hypholoma sublateritium*	Cinnamon cap	58 (56)	− 3.09	− 3.09	0.07	− 3.23	− 2.95	− 0.040	− 0.041	0.031	− 0.102	0.019	89	77	102	− 6.0
		*Lepista nuda*	Wood blewit	14 (13)	− 2.52	− 2.51	0.14	− 2.77	− 2.24	− 0.074	− 0.075	0.041	− 0.156	0.000	83	68	98	− 3.6
		*Lyophyllum decastes*	Fried chicken mushroom	75 (30)	− 4.16	− 4.16	0.07	− 4.30	− 4.02	− 0.037	− 0.038	0.033	− 0.100	0.025	90	78	103	− 6.3
		*Pholiota microspora*	Nameko (ja)	41 (38)	− 3.05	− 3.05	0.12	− 3.29	− 2.81	− 0.086	− 0.088	0.046	− 0.183	0.001	80	64	98	− 3.1
		*Pholiota squarrosa*^1)^	Shaggy scalycap	31 (12)	− 4.22	− 4.22	0.11	− 4.44	− 4.00	− 0.064	− 0.064	0.043	− 0.151	0.019	84	69	102	− 4.1
Wild plants	Bamboo shoot	*Phyllostachys heterocycla, P* *bambusoides, P. nigra* var *henonis* etc.^2)^	Bamboo shoots, shoot	300 (198)	− 3.90	− 3.90	0.04	− 3.98	− 3.83	− 0.121	− 0.121	0.017	− 0.156	− 0.088	74	68	80	− 2.3
	Deciduous tree	*Aralia elata*	Taranome Japanese angelica-tree, shoots	124 (81)	− 3.67	− 3.67	0.05	− 3.77	− 3.57	− 0.047	− 0.048	0.025	− 0.097	0.001	88	78	98	− 5.2
		*Castanea crenata*	Japanese chestnut, nuts	123 (53)	− 3.94	− 3.93	0.09	− 4.13	− 3.75	− 0.036	− 0.037	0.039	− 0.113	0.037	90	75	106	− 6.5
		*Eleutherococcus sciadophylloides*	Koshiabura (ja), shoot	56 (55)	− 2.52	− 2.52	0.07	− 2.66	− 2.37	0.014	0.014	0.034	− 0.051	0.080	101	87	118	77.6
		*Vaccinium oldhamii*	Oldham blueberry, berry	43 (14)	− 4.39	− 4.39	0.10	− 4.59	− 4.19	− 0.073	− 0.074	0.046	− 0.164	0.013	83	67	101	− 3.6
		*Viburnum dilatatum*	Linden viburnum, berry	20 (10)	− 4.22	− 4.22	0.13	− 4.48	− 3.96	− 0.068	− 0.071	0.055	− 0.185	0.032	83	64	105	− 3.8
	Perrenial fern	*Matteuccia struthiopteris*	Ostrich fern, shoot	86 (50)	− 4.03	− 4.03	0.06	− 4.16	− 3.92	− 0.011	− 0.012	0.032	− 0.076	0.051	95	82	110	− 14.4
		*Osmunda japonica*	Asian royal fern, shoot	78 (73)	− 3.20	− 3.20	0.06	− 3.32	− 3.08	− 0.025	− 0.025	0.031	− 0.088	0.038	92	80	107	− 8.6
		*Pteridium aquilinum*	Western bracken fern, shoot	597 (325)	− 4.09	− 4.09	0.03	− 4.14	− 4.03	0.016	0.016	0.013	− 0.008	0.041	101	96	107	46.9
	Perrenial herb	*Aralia cordata*	Japanese spikenard, shoots	478 (122)	− 4.51	− 4.51	0.04	− 4.59	− 4.45	− 0.021	− 0.021	0.018	− 0.057	0.014	93	86	101	− 9.6
		*Artemisia indica* var *maximowiczii*	Japanese mugwort, shoots	64 (25)	− 4.37	− 4.37	0.08	− 4.53	− 4.21	− 0.052	− 0.053	0.038	− 0.130	0.021	87	72	103	− 4.8
		*Eutrema japonicum*	Japanese horseradish, shoot	12 (10)	− 4.09	− 4.09	0.18	− 4.45	− 3.74	− 0.073	− 0.074	0.052	− 0.181	0.023	83	64	103	− 3.6
		*Parasenecio delphiniifolius*	Momijigasa (ja), shoot	104 (64)	− 4.07	− 4.07	0.06	− 4.19	− 3.96	− 0.052	− 0.052	0.028	− 0.106	0.002	87	77	98	− 4.8
		*Petasites japonicus* (leaf stalk)	Japanese butterbur, leaf stalk	746 (168)	− 4.60	− 4.60	0.03	− 4.67	− 4.54	− 0.039	− 0.039	0.014	− 0.066	− 0.013	89	84	95	− 6.2
		*Petasites japonicus* (scape)	Japanese butterbur, scape	146 (74)	− 4.10	− 4.10	0.05	− 4.21	− 4.00	− 0.061	− 0.061	0.025	− 0.111	− 0.014	85	76	95	− 4.2
		*Synurus pungens*	Oyamabokuchi (ja), shoot	38 (21)	− 4.08	− 4.08	0.10	− 4.27	− 3.89	− 0.001	− 0.001	0.043	− 0.083	0.083	97	81	118	− 26.5

^1)^*P. squarrosa* is reported as poisonous. Also, it may contain similar edible species.

^2)^Among bamboo shoots, *P. heterocycla* (moso-bamboo) is the most popular in Japan but may contain multiple species.

^3)^"(ja)" indicates a Japanese name.

^4)^Number of parentheses indicates number of samples of which radioceisum concentration was detected.

^5)^Positive and negative values indicate doubling time and half life, respectively. These values are calculated from posterior median λsp considering radioactive decay of ^137^Cs (half life, 30.17 year).

**Figure 2:**
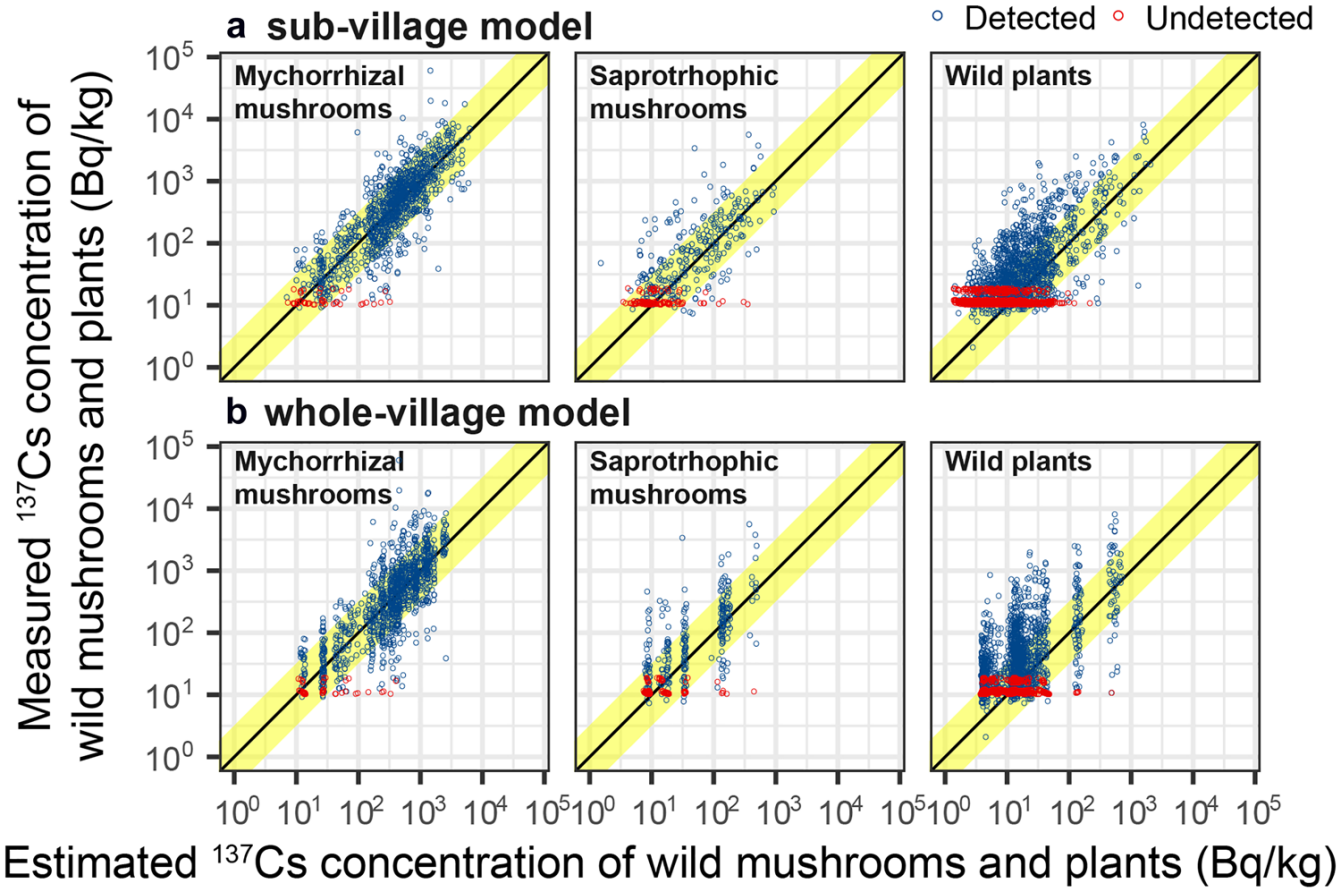
Comparison between measured and estimated ^137^Cs activity concentration of wild mushrooms and plants. For estimated values, each sample’s posterior median using the sub-village model (**a**, upper) and the whole village model (**b**, lower) is shown on the horizontal axis. The results were separately plotted for mycorrhizal mushrooms (left), saprotrophic mushrooms (center), and wild plants (right). The scales of both axes are logarithmic. Red symbols indicate the measured values were less than detection limits that were plotted as measured values. The points on the yellow bands indicate that the estimated values fell within 10-folds variation from the measured values. Table S3 shows the number and percentage of detected data falling into the bands.

We obtained posterior results of each parameter by Bayesian estimation. Using these parameters, we estimated the ^137^Cs concentration of each specimen based on models and compared it with the measured value (Fig. 2). In Fig. 2a, the estimated median from the sub-village model was plotted, and results of whole village model were shown in Fig. 2b. For additional comparison, the plots were separately shown for mycorrhizal mushrooms, saprotrophic mushrooms, and wild plants. Among mushroom species, mycorrhizal fungi form symbiotic relationships with plants and uptake carbon via plant roots, while saprotrophic fungi decompose dead or dying organic matter for their nutrition. In both models, the estimated and measured values were distributed around the 1:1 line. The deviation from the 1:1 line was slightly smaller in the sub-village model. Among detected samples (blue symbols), 71% (1926/2700) and 67% (1801/2700) of the estimated values from the sub-village model and the whole village model were within ten times the range of the measured values (yellow belts in Fig. 2), respectively. The difference was significantly different (*p* < 0.001, chi-squared test, Table S3).

Table 2 shows the posterior distributions of individual error (*σ*) and the hierarchical parameters. The sigma (*σ*), which indicates the magnitude of the deviation from the mean estimated by the model (*μ*_sample_), was 0.51 for the sub-village model and 0.57 for the whole village model. The 95% confidence intervals for *σ* did not overlap between the two models (0.50–0.53 in the sub-village model and 0.55–0.59 in the whole village model); this indicates that the variability of the concentration becomes smaller when the sampling points can be specified at the sub-village level. The variability parameters of the concentration characteristics among species (*σ*_sp_) were 0.81 and 0.83, which were larger than the individual error.

**Table 2:** Posterior distribution of parameters of two models.

Model	Parameter	Median	Mean	SD	CI2.5%	CI97.5%
Sub-village model	*μ*_sp_	− 3.41	− 3.41	0.13	− 3.68	− 3.14
	*μ*_*λ*sp_	− 0.022	− 0.022	0.012	− 0.046	0.000
	*σ*	0.51	0.51	0.01	0.50	0.53
	*σ*_loc_	0.29	0.29	0.04	0.22	0.37
	*σ*_sp_	0.83	0.84	0.10	0.67	1.05
	*σ*_λsp_	0.057	0.058	0.010	0.041	0.081
Whole village model	*μ*_sp_	− 3.56	− 3.56	0.13	− 3.82	− 3.30
	*μ*_*λ*sp_	− 0.002	− 0.002	0.011	− 0.025	0.019
	*σ*	0.57	0.57	0.01	0.55	0.59
	*σ*_sp_	0.81	0.82	0.10	0.65	1.05
	*σ*_λsp_	0.049	0.050	0.010	0.033	0.072

### Characteristics of radiocesium concentration in mushroom and edible plant species

**Figure 3:**
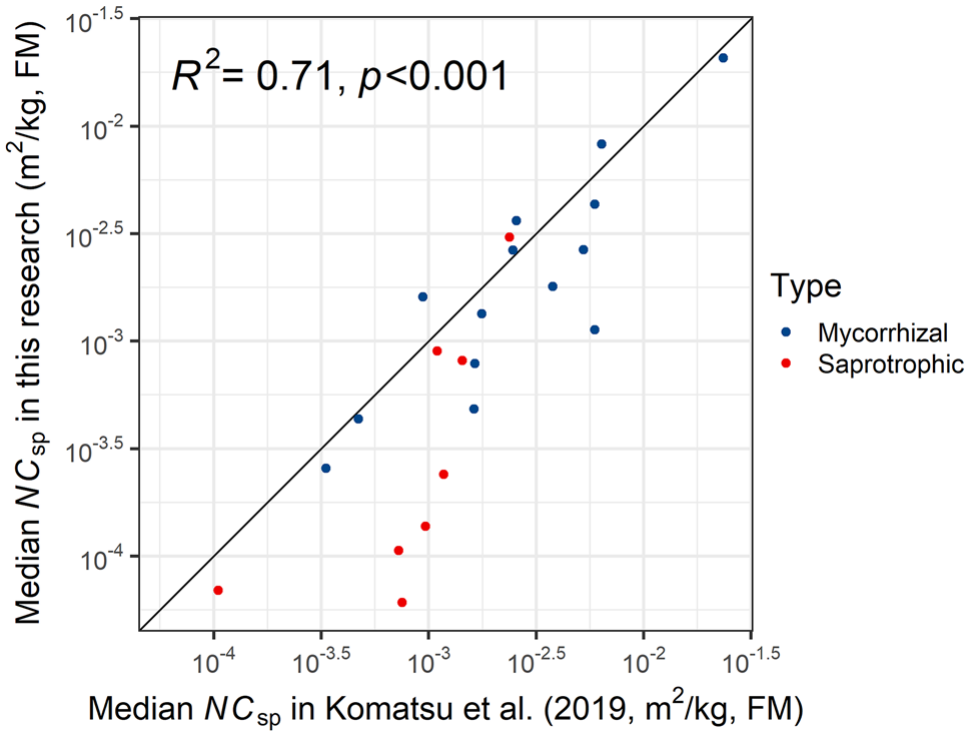
Comparison of normalized concentration parameters of mushroom species (*NC*_sp_). Only the mushroom species common to Komatsu et al.^22^ and in this study were considered. The scales of both axes are logarithmic.

Table 1 shows the calculated posterior results for concentration (*NC*_sp_) and temporal trends (*λ*_sp_) for each species. Here, the effective annual change ratio (*R*_eff_sp_) and half-lives (doubling time) were calculated using *λ*_sp_. The median and mean values of *NC*_sp_ differed by more than 100-fold (up to 845 times between *Cortinarius salor* (*C. iodes*) and *Petasites japonicus* (leaf stalk)) among species. *NC*_sp_ of mushroom species in this study was compared with *NC*_sp_ of previous research^22^ in Fig. 3, and with Cs_ess_ from Tagami and Uchida^31^ in Supplementary Fig. S1. Both comparisons showed a positive and high correlation coefficient (*R*^2^ = 0.713, 0.764, respectively).

**Figure 4:**
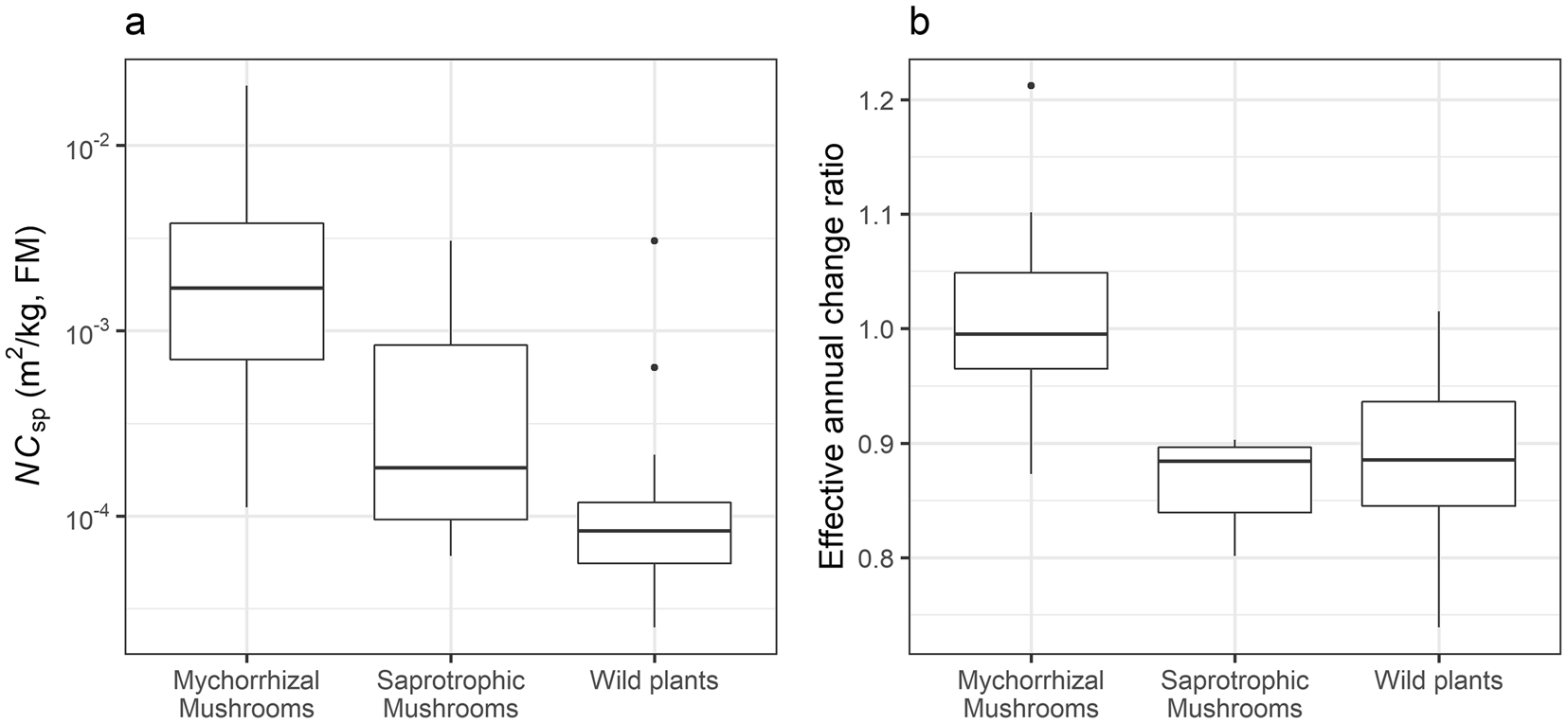
Boxplots of species’ concentration parameters according to type. (**a**) Normalized ^137^Cs concentration of species (*NC*_sp_, log scale) and (**b**) effective annual change ratio of ^137^Cs concentration (*R*_eff_sp_). The unit for *NC*_sp_ is m^2^/kg fresh matter (FM).

Figure 4 shows the median posterior *NC*_sp_ and *R*_eff_sp_ values by type presented as boxplots. The *NC*_sp_ values of mycorrhizal mushrooms (Fig. 4a) are the highest, followed by saprotrophic mushrooms, and the lowest values are for wild plants. However, even among the same types, there was a large variation in the *NC*_sp_ values. For example, in mycorrhizal fungi, the median *NC*_sp_ values ranged 189-fold between *C. salor* and hygroscopic earthstar (*Astraeus hygrometricus*). Most wild plants had *NC*_sp_ values of < 10^−3^, but only koshiabura (*Eleutherococcus sciadophylloides*) had significantly higher values (3.0 × 10^−3^ m^2^/kg, FM). In addition, stalk and scape of Japanese butterbur (*Petasites japonicus*) were separately analyzed, and the *NC*_sp_ of butterbur scape was 3.2 times higher than the stalk.

The *R*_eff_sp_ values of mycorrhizal mushrooms (Fig. 4b) are higher than the other types, with half of medians (8 of 16) greater than one. In particular, the *R*_eff_sp_ values of *Sarcodon aspratus* (“Kotake” in Japanese) are clearly greater than 1 (the median and lower 95% confidence interval value are 1.21 and 1.16, respectively, Table 1). Meanwhile, the *R*_eff_sp_ values of most saprotrophic mushrooms and all wild plants are less than 1 (22 of 24), indicating a decreasing trend of ^137^Cs concentrations with time.

### The characteristics of the regional parameter

**Figure 5:**
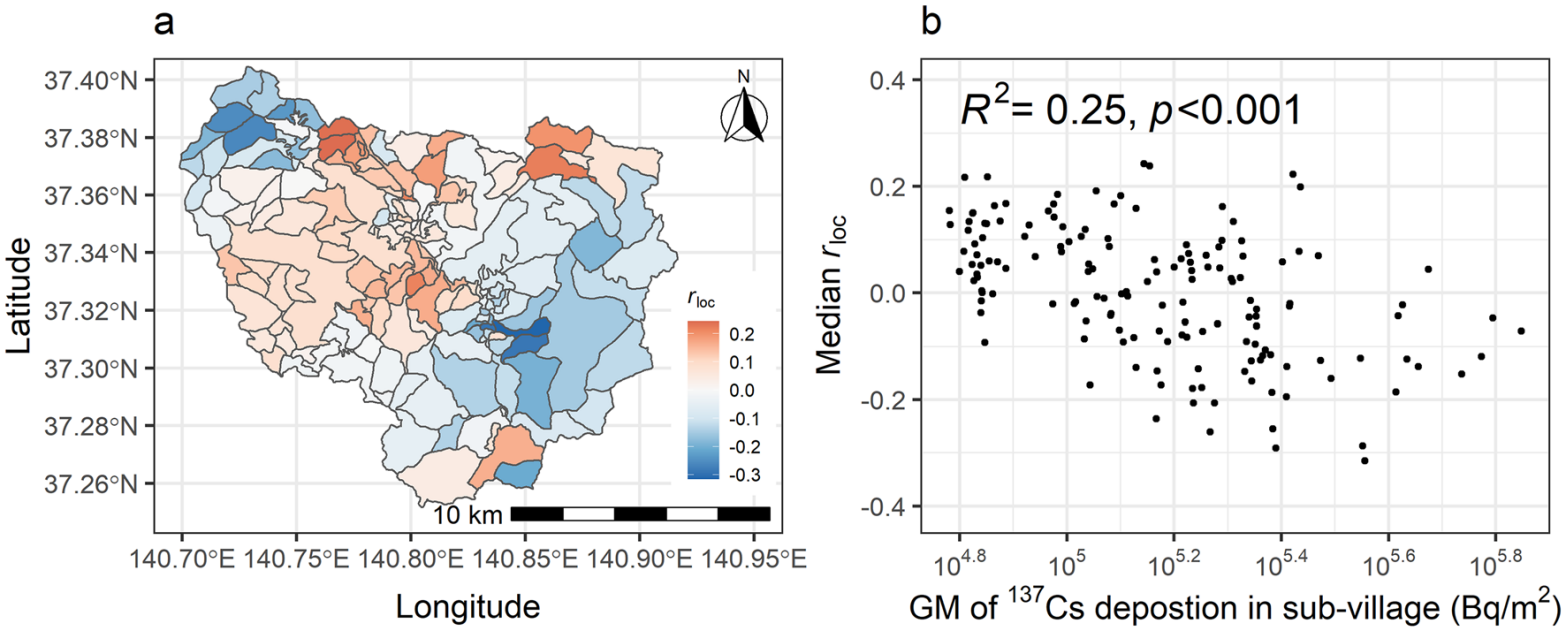
(**a**) The map of median posterior spatial parameter affecting on concentration (*r*_loc_). (**b**) Relationship between ^137^Cs deposition and median *r*_loc_ of each sub-village. The sub-village boundaries were created from a hand-drawn map obtained from the administrative office. The map was created using R version 4.1.0^29^ and ggplot2 package^30^.

The spatial distribution of the median *r*_loc_ of the regional parameters affecting the radioactivity of samples is shown in Fig. 5a. The *r*_loc_ tended to be higher in the village’s central and western part. However, the area bordering the northwestern village boundary and the southeastern area had lower *r*_loc_. Because the model was expressed as a sum of normalized logarithmic parameters, the value of *r*_loc_ to the 10th power (10^*r*loc^), which ranged 3.61 times as maximum, had the effect of adjusting the concentration characteristics of each sub-village from the amount of ^137^Cs deposition (*D*_loc_). When estimated *r*_loc_ and the geometric mean of ^137^Cs deposition of each sub-village was plotted, there was a significant negative correlation (*R*^2^ = 0.253, *p* < 0.001, Fig. 5b). In Fig S2, we show a map of the product of ^137^Cs deposition (*D*_loc_) and *r*_loc_ to the 10th power (*D*_loc_ × 10^*r*loc^) for each sub-village. The multiplied values indicate the adjusted effect on the sample ^137^Cs concentration at the sub-villages. Although this figure is similar to Fig. 1b, the values increased at the central area, and the difference of the colored value is slightly reduced between sub-villages; the maximum ranges of the adjusted concentration effect (*D*_loc_ × 10^*r*loc^, Fig. S2) and ^137^Cs deposition (*D*_loc_, Fig. 1b) are 10.5 and 11.7, respectively.

## Discussion

The radiocesium levels of mushroom species in our study agree with those of previous studies (Fig. 3, Fig. S1). Komatsu et al.^22^ analyzed the food monitoring data of wild mushrooms obtained at various municipalities in eastern Japan after the FDNPP accident and evaluated *NC*_sp_ as in this research. Tagami and Uchida^31^ analyzed radiocesium concentration of wild mushrooms from previous literature and monitoring data and evaluated ^137^Cs in mushrooms originating before the FDNPP accident, such as nuclear bomb tests. Each of the two results is a generalization of radiocesium concentration characteristics in wild mushrooms, but it was necessary to confirm the results’ universality using another data set. Because the results obtained in one small area (Kawauchi Village) demonstrated a high positive correlation with the results of data collected over a wide area, it was confirmed that each wild mushroom species’ concentration characteristics exist systematically in Japan. Although the concentration characteristics of wild plants were not validated sufficiently in this research, koshiabura, which has the highest *NC*_sp_ among wild mushrooms, has the largest number of municipalities with shipping restrictions (113 in March 2021), while taranome (*Aralia elata*) and Asian royal fern (*Osmunda japonica*), which have a moderately higher *NC*_sp_ of 10^–3^, have the second largest number of restricted municipalities after koshiabra (44 and 22, respectively)^10^. Therefore, there is a correspondence between the concentration characteristics and the number of cities, towns, and villages with shipping restrictions. As with wild mushrooms, each species of wild plants’ concentration characteristics is universal.

A comparison of the trends of *NC*_sp_ and *R*_eff_sp_ values of the ecological groups shows clear group differences in these parameters (Fig. 4, Table 1). The *NC*_sp_ values reflect the ^137^Cs concentration trend for each species, normalized for the effects of deposition, sampling location (sub-village in this research), and sampling date (year). The fact that the radiocesium contents in mycorrhizal mushrooms are generally higher than saprotrophic mushrooms has been widely accepted by research conducted after both Fukushima and Chernobyl nuclear power plant accidents^22,32,33^. Yoshida and Muramatsu^34^ hypothesizes that differences in their mycelium distribution zones would explain differences in radiocesium concentrations among ecological types of mushroom species. Mycorrhizal fungi generally develop their mycelium in soil, while the saprotrophic family grows mycelium on dead wood and fallen leaves for nutrient absorption. It is known that most of the radiocesium is transferred to the surface layer of mineral soil with time after the accident. In a survey conducted by Imamura et al.^5^ in a cedar forest in Kawauchi Village, 30.7%, 31.0% and 24.7% of ^137^Cs was distributed in trees, organic layer, and the mineral soil layer in the summer of 2011, respectively. However, the ^137^Cs proportions in the trees and organic layer decreased to 4.0% and 7.8%, respectively, whereas those of mineral soil increased significantly to 88.2%. In mineral soils of forest ecosystems, ^137^Cs has been demonstrated to remain in the surface (0–5 cm depth) mineral surface layer (65%–91% of the total ^137^Cs in the mineral soils). The hypothesis that radiocesium concentration in mycelium’s habitat affects the mushrooms’ concentration could explain the differences in concentration between types. Future research is required to quantitatively evaluate the relationship between radiocesium concentration and the mycelium distribution among species and types.

The higher radiocesium contents in mushrooms than in wild plants are thought to be due to differences in their ability to absorb nutrients. Yoshida and Muramatsu^35^ compared the concentration of various metal elements in fungi and plants with the radiocesium concentration, and they found that mushrooms had higher potassium (K) concentrations and radioactive and stable cesium (^137^Cs and ^133^Cs) concentrations than plant leaves. However, radiocesium concentrations are variable even among wild plant species, and for example, *Eleutherococcus sciadophylloides* (“Koshiabura” in Japanese) is close to the values of mycorrhizal mushrooms (Table 1). Kiyono and Akama have shown that radiocesium concentration in koshiabura is related to radiocesium deposition in the litter^19,36^. These results indicate that koshiabura grows its roots at the surface and can absorb radiocesium more efficiently. Yamaji et al.^37^ suggested that siderophores produced by endophytic root bacteria in koshiabra promote absorption by desorption of ^137^Cs in the rhizosphere. It would be desirable to clarify the mechanisms causing intraspecific variation in concentration referencing the present results.

Among species, the time-dependent parameter, *R*_eff_sp_, also showed different trends between ecological groups (Fig. 4b, Table 1). The ^137^Cs concentrations in samples were decay-corrected at the time of the accident (March 11th, 2011) and the physical decay of ^137^Cs (30.17 years) was not accounted for in the *λ*_sp_ calculation. On the other hand, *R*_eff_sp_ involves physical decay of ^137^Cs, and *R*_eff_sp_ values greater or less than one indicate increasing or decreasing trends, respectively. Therefore, the trends indicate that the ^137^Cs concentrations in the half of mycorrhizal species are increasing, while those of saprotrophic mushrooms and wild plants are decreasing. Some of the differences in the annual trends among these groups can be explained by the temporal changes in radiocesium concentrations in their respective substrates. The increasing ^137^Cs concentrations of mycorrhizal fungi corresponds to the increasing concentration of ^137^Cs in the soil, and the decreasing ^137^Cs concentrations of saprotrophic fungi corresponds to the decreasing concentrations of ^137^Cs in litter and dead wood^5^. In particular, kotake mushrooms demonstrated a clear increasing trend. Yamada et al. reported the possibility of accumulation of ^137^Cs in mycelium because many of the mycorrhizal mushrooms with high concentrations retained a large amount of ^137^Cs originating from events prior to the Fukushima accident^38^. It is necessary to confirm whether such retention of ^137^Cs in mycelium is actually occurring.

On the other hand, the decreasing ^137^Cs concentration in wild plants that uptake nutrients from mineral soils cannot be explained by the increasing levels of ^137^Cs in their substrates. Manaka et al.^39^ shows that the proportions of exchangeable radiocesium in organic layer and mineral soil layer were high in 2011 (6–10%), immediately after the accident 2011 and then decreased (2–4%). The distribution of radiocesium in the forest changes over time, and the accessibility of radiocesium changes too. Experiments demonstrated that mycelium has the ability to solubilize Cs adsorbed in soil^40^. However, plants are not able to solubilize such Cs adsorbed in soil; therefore, the decrease in accessibility of radiocesium over time in forest soils was considered to be responsible for decrease in the concentration of wild plants.

The concentration characteristics of the species varied greatly (Table 1), confirming that it is extremely important to consider the effect of species in estimating the concentration of wild mushrooms and plants. On the other hand, from comparison results, more detailed information of sampling points would improve the estimation accuracy of radioactivity in wild mushrooms and plants when collection points could be obtained (Fig. 2 and Table 2). In Fig. S4, the normalized concentration, which was calculated by dividing the ^137^Cs concentration of wild mushrooms and plants by the amount of ^137^Cs deposition, is colored according to the *r*_loc_ values of the sampled sub-villages. This visualization shows that samples collected at sub-villages with higher *r*_loc_ values tended to exhibit higher normalized concentrations within a given species. The 95% confidence intervals for ^137^Cs concentration in the samples from the normal distribution and the median of posterior *σ* (Table 2) was estimated to be a factor of 103 (= 10^0.51×2×1.96^) for the sub-village model and a factor of 171 (= 10^0.57×2×1.96^) for the whole-village model. This result simply indicates that information of sample collection site in each village would reduce sample variability by 40%, assuming that the concentration varies among sub-villages according to the deposition (*D*_loc_) adjusted by *r*_loc_. Such an improvement in estimation accuracy is expected to contribute to the evaluation of the internal dose according to ingestion and collection behavior.

However, the sub-village model, which takes into account species and site effects, still showed large errors. This result suggests variation in concentration among specimens of the same species collected within a few square kilometers of a sub-village. Previous studies have shown that radiocesium concentrations in wild mushrooms of a given species can fluctuate within an even smaller area. Dahlberg et al.^41^ investigated intraspecific variability of ^137^Cs concentrations of one mushroom species (*Suillus variegatus*) from seven sites within 1 km^2^ area. Almost 40% of the variation in ^137^Cs levels was accounted for by the sites, and the other 60% was accounted for within sites. Komatsu et al.^42^ investigated ^137^Cs concentration of *Boletus hiratsukae* at two plots about 300 m apart and showed that the geometric mean of the mushroom concentrations at the two sites differed by a factor of 3.5, and that even within a site of about 200 m^2^ the mushroom concentrations differed by a factor of nine. Location information of greater accuracy is expected to reduce the error in estimating the concentration of specimens. However, there may be a limit to the use of location information alone because it is affected by variation in the amount of radiocesium deposited in the forest and the environment. The factors governing variation in concentration within a given forest must be explained.

The location parameters affecting concentration (*r*_loc_) are spatially biased (Fig. 5a). Radiocesium concentration in wild mushrooms and trees is known to be affected by deposition and by soil properties such as exchangeable K concentration, pH, and clay mineral content^42–45^. Therefore, one possibility is that the characteristics of the soil may have caused the deviation between the *r*_loc_ concentration and the amount of deposition. However, *r*_loc_ was higher in the central area where deposition was lower, and there was a significant negative correlation between log_10_*D*_loc_ and *r*_loc_ (Fig. 5b). One possible explanation for this relationship is that the deposition across the forest was not sufficiently evaluated by aircraft monitoring. The central area is relatively flat along valley floors, and it has farmland and residential areas. In general, radioactive cesium deposited on farmland and residential areas is more likely to move than forests^46^. Therefore, the deposition of ^137^Cs on farmlands and residential areas is usually lower than that on nearby forests. The ^137^Cs deposition data used in this research have a 250-m resolution, having been measured from helicopters piloted at an altitude of approximately 300 m^47^. Therefore, the amount of cesium deposited in forests in the central area may have been underestimated due to the mixing effect of other land covers, and this may have been recognized as a difference in *r*_loc_.

This study has data quality limitation because of the collection of the data by residents. The data may include some ambiguous reporting; for example, some locations of collection sites may actually be the collector’s residence. This is because it is sometimes difficult for the collectors to report the exact location of collection within the vast forested areas. In addition, for expensive mushrooms such as matsutake (*Tricholoma matsutake*), which can fetch some hundreds of dollars per kilogram, there is a risk that collection locations will not be accurately reported. If samples with high radiocesium activity were collected at the outer side of the village (with high amounts of radiocesium deposition) but were reported as having been collected in the central area (with rates of lower deposition), then the *r*_loc_ value of the central area will be overestimated as shown in Fig. 5b. However, Bayesian analysis of the data set that excluded matsutake shows little change in the negative correlation between *r*_loc_ and *D*_loc_ (Supplementary Fig. S3). Thus, uncertainty in the sampling location did not have a significant effect on *r*_loc_. There was a large number and variety of samples distributed widely throughout the village, indicating that our results are robust. Therefore, the data originating from samples obtained by the residents are worth analyzing and are likely to provide valuable research conclusions.

## Conclusion

We showed the radiocesium concentration’s trend in wild mushrooms and wild edible plants using inspection data of food. For mushrooms, since the values were close to those of previous studies, the estimated values of species were considered to be widely applicable, at least in Japan. In addition, this study shows that the data from samples collected by residents can be used to analyze the concentration characteristics of species and regions. In the future, it will be possible to use such data for internal exposure assessment by collecting wild food consumption data that takes into account regional and individual differences. Furthermore, such results may also encourage sustained sampling efforts by citizens. The inspection of food brought in by residents is already widely conducted at municipalities in Fukushima Prefecture. Should this continue, then we expect further development of participatory research by citizens.

It is said that radiocesium in forests approaches an equilibrium state with time^13^. However, the radiocesium concentrations in some species, such as *Sarcodon aspratus* (one of the popular and economically valuable edible mushrooms in the area) are still trending upward and are not in equilibrium. It is necessary to continue monitoring to show the prospect of concentration changes.

## Materials and methods

### Site information

We collected radioactivity data of wild mushrooms and wild edible plants from inspection results of specimens brought in by residents in Kawauchi Village, which is located 12–30 km away from the FDNPP (Fig. 1). Kawauchi Village is considered small, with an area of 197.4 km^2^, and a population of about 2500 (2820 in 2010 and 2518 in 2021)^48^. It is located in the middle of the Abukuma Highlands, where the elevation ranges from 270 to 1,192 m above the sea level. It has a forest coverage of 89.0%, which is higher than the average for Fukushima Prefecture (71%) and Japan as a whole (69%)^49^. ^137^Cs deposition in the village ranged from 42 to 960 kBq/m^2^ in 2011, estimated from an aircraft monitoring^28^. Before the accident, its residents were accustomed to gathering wild foods, such as wild edible mushrooms, plants, mammals, and wild honey^50^; many have been brought in for inspection. Information on collection areas of sub-village levels, called “Ko-aza” in Japanese, is also recorded. For these reasons, we thought that the data of the brought in inspection in Kawauchi Village would possess high value as data for inter-species and inter-region analysis on the wild mushrooms and edible plants’ radioactivity concentrations.

### Radioactivity data of mushrooms and wild plants

Fukushima Prefecture sets up a system for each municipality to inspect radioactivity in vegetables and mushrooms consumed by residents, and Kawauchi Village started its inspection program in May 2012. Simple inspection machines are set up at public facilities, and inspections are conducted upon application by residents. In Kawauchi Village, the location of samples inspected was requested at the sub-village level. The inspection results were regularly reported in the village newsletter, along with the inspection date, inspected food, and collection location. The data compiled from May 2012 to March 2020 was provided to us through the village officials. Orita et al. analyzed the same inspection data of agricultural products in Kawauchi Village^24^. They used 7668 food data from April 2013 to December 2014, including 1986 wild plants and mushrooms data for internal radiation exposure assessment. Some of their data overlap with the data used in our analyses.

### System of monitoring radioactivity in Kawauchi Village

Kawauchi Village started the brought in inspection in May 2012, and there is a maximum of eight inspection stations and currently three stations managed by residents. In the inspection sites, there are four types of NaI (Tl) or CsI (Tl) scintillation detectors. The machine names are Triathler Becquerel Finder (Hidex, Oy, Finland), Captus-3000A (Capintec, NJ), CAN-OSP-NAI (Hitachi Aloka, Tokyo, Japan), and FD-08Cs1000-1 (X-Ray Technology, Osaka, Japan). Table S4 shows the specifications of the machines^51–53^. All instruments have been confirmed to meet the radiocesium screening method requirements for food^53^. Among these machines, FD-08Cs1000-1 can measure radioactivity non-destructively, and the others conduct destructive measurements. The sample weight is approximately 500 g, and the counting time is 30 min. FD-08Cs1000-1 outputs the summed concentration of the two radiocesium nuclides (^134^Cs and ^137^Cs), and its detection limit is 10 Bq/kg (for total ^134^Cs + ^137^Cs). Each of the other three machines separately outputs the concentrations of ^134^Cs and ^137^Cs, and the detection limit is 10 Bq/kg for each radionuclide. Energy calibrations and background checks were performed daily, and the accuracy was periodically verified with brown rice whose radiocesium concentration was verified by calibrated high-purity Germanium (HPGe) detectors installed in the Fukushima Nuclear Center^49^. Table S4 shows the results of quality control using brown rice.

### Data preparation of radioactivity of samples

From the radioactivity data of wild mushrooms and plants, we picked up data that met the following criteria;

- Data have information of sampling location at sub-village levels
- Items that are not confirmed to be cooked products such as “boiled” or “dried.”
- Species with more than ten samples in which radiocesium was detected.

In cases where mushrooms and wild plants were given in dialects, we confirmed the species’ names with residents. The names of the species were determined from the Japanese names of the items, but in some cases, it was not possible to distinguish between *Cortinarius salor* (“Murasakiaburashimejimodoki” in Japanese) and *C. iodes* (“Murasakiaburashimeji”), considered to be closely related species, so the two were mixed for analysis. The leaf stalk and scape of *Petasites japonicus* (Japanese butterbur) are called “Fuki” and “Fukinotou” in Japanese, respectively, and are registered separately. Therefore, despite being the same species, they were distinguished in the analysis. In this data, there were not sampling date but measurement date. Therefore, the date of measurement and sample collection were assumed to be the same.

The ^137^Cs concentration results were used in the model analysis. The reason for not using the^134^Cs concentration among the measured values is explained in the subsection of “Bayesian estimation”. ^137^Cs concentrations were decay-corrected to March 11th, 2011 for comparison with Komatsu et al. (2019). Based on the assumption that the ^134^Cs/^137^Cs ratio at the time of the accident was one^54^, the summed concentration of ^134^Cs and ^137^Cs concentration taken by FD08-Cs1000-1 was converted to a ^137^Cs concentration, which was decay-corrected to March 11th, 2011, using the following equation;

$${}^{137}C{s}_{2011/03/11}=tC{s}_{\mathrm{sampling}\_\mathrm{day}}*\frac{{0.5}^{dy/30.17}}{{0.5}^{dy/2.065}+{0.5}^{dy/30.17}}$$

In this equation, *dy* indicates the period from March 11th, 2011, to the date of measuring, and it is expressed by decimal years.

### Sub-village (“Ko-aza”) boundary map of Kawauchi Village

Kawauchi Village comprises eight administrative communities (called “Oh-aza” in Japanese), which are further subdivided into small administrative units known as “Ko-aza”. Here, we refer to these small administrative units as sub-villages. We obtained a sub-village map from the administrative office. The printed map was originally drawn by hand and had been used for village administration. To create a polygon shapefile of the map, we digitized it by scanning, geo-rectifying, and digitizing using GIS software in TNTmips v2014 (MicroImages, Inc, NE) and ArcGIS 10.3 (Esri, Inc, CA). We used this map to associate land names with monthly radioactivity data from samples and to estimate sample collection locations.

### Deposition data

For the ^137^Cs deposition data of this area, we used 250 m grid deposition data measured by the Ministry of Education, Culture, Sports, Science and Technology^28,55^ and then corrected by Kato and Onda^26^. We computed the geometric mean value of ^137^Cs deposition within each sub-village polygon. The ^137^Cs deposition is also decay-corrected to March 11th, 2011.

### Bayesian estimation

We constructed a Bayesian model partially modified from Komatsu et al.^22^ to estimate ^137^Cs concentration (^137^Cs_sample_). The model is based on the Gonze and Calmon’s concept of normalized concentration (*NC*) as expressed by:

$$NC= \frac{Cs}{D}$$

where *D* indicates the radiocesium deposition amount based on the aircraft monitoring. Then the above equation is transformed and logarithmized to yield;

$$\mathrm{log}Cs=\mathrm{log}NC+\mathrm{log}D$$

In this expression of the model equation, we further assumed that the logartihm of *NC* encompassed the summed effects of species identity, collection date, and collection site, and that the logarithm of *NC* was normally distributed around the estimated mean as per the following equations;

$$\begin{array}{l}{\text{log}}_{10}{\hspace{0.17em}}^{137}C{s}_{\mathrm{sample}} \sim Normal({\mu }_{\mathrm{sample}},\sigma )\\ {\mu }_{\mathrm{sample}} ={\text{log}}_{10}N{C}_{\mathrm{sp}}+{\lambda }_{\mathrm{sp}}Y+{\text{log}}_{10}{D}_{\mathrm{loc}}+{r}_{\mathrm{loc}}\\ {\text{log}}_{10}N{C}_{\mathrm{sp}} \sim Normal({\mu }_{\mathrm{sp}},{\sigma }_{\mathrm{sp}})\\ {\lambda }_{\mathrm{sp}} \sim Normal({\mu }_{\mathrm{\lambda sp}},{\sigma }_{\mathrm{\lambda sp}})\\ {r}_{\mathrm{loc}} \sim Normal(0,{\sigma }_{\mathrm{loc}})\end{array}$$

where *NC*_sp_, *λ*_sp_, *D*_loc_ and *r*_loc_ indicate characteristics of concentration of species, temporal trends of species, ^137^Cs deposition of each sub-village area and effects of sub-village on concentration, respectively. *r*_loc_ is a parameter with zero mean that represents the deviation of the concentration effect from the expected value based on the deposition (*D*_loc_) value at the point of collection. These parameters except *D*_loc_ were obtained from hierarchically sampled from normal distribution with hierarchical parameters (*μ*_sp_, *σ*_sp_, *μ*_λsp_, *σ*_λsp_, *σ*_loc_). Additionally, *r*_loc_ was sampled using the Intrinsic Conditional Auto-Regressive (Intrinsic CAR) model^56^, which is one of the models considering spatial auto-correlation. For samples whose measured radiocesium concentrations were below the detection limit, radiocesium concentration values were estimated by a censoring distribution in which the detection limit was treated as the upper bound^57^. This model was defined as the “sub-village model” for this research. This model is similar to model 6 in Komatsu et al.^22^ but differs in that their previous model takes into account ^134^Cs values and differences between ^134^ and ^137^Cs values. Komatsu et al. evaluated the regional trend in the difference between^134^Cs and ^137^Cs concentrations across eastern Japan because ^137^Cs originating from nuclear bomb tests before the FDNPP accident was detected in wild mushrooms sampled in the northern and southern parts of eastern Japan, which are far from the FDNPP and received less deposition from the accident (< 10 Bq/m^2^ according to aircraft monitoring). However, in Kawauchi Village, the amount of ^137^Cs deposition because of the FDNPP accident (42–960 Bq/m^2^ in Kawauchi Village^26^) was larger than the amount of ^137^Cs deposition from nuclear tests (< 10 kBq/m^2^ in Japan^58^), and it is difficult to evaluate differences between ^137^Cs and ^134^Cs concentrations of each specimen. Therefore, our analysis used only ^137^Cs values given the longer half-life of that isotope.

Alternatively, for the comparison, we also used the “whole village model,” in which *r*_loc_ was not taken into account and the geometric mean of the ^137^Cs deposition in the village (*D*_village_) was used for the calculation.

$${\mu }_{\mathrm{sample}}={\text{log}}_{10}N{C}_{\mathrm{sp}}+{\lambda }_{\mathrm{sp}}Y+{\text{log}}_{10}{D}_{\mathrm{village}}$$

For the Bayesian estimation, we used WinBUGS ver 1.4.3^57^. In the calculation, we set burn-in, burn-out, chain, and thin as 10,000, 20,000, 3, and 10, respectively. As a result, we gained 3000 posterior results for each parameter.

The effective annual change ratio according to species (*R*_eff_sp_) was calculated based on the radiological decay of ^137^Cs (half-life: 30.17 years) and using *λ*_sp_ as follows:

$${R}_{\mathrm{eff}\_\mathrm{sp}}={10}^{{\lambda }_{\mathrm{sp}}}{0.5}^{1/30.17}$$

### Limitation

The date of collection of each sample was not registered, and the substitution of the measurement date for the collection date may cause uncertainty in the analysis of temporal trends. However, the measurement dates were reported in a concentrated manner for each species (Fig. S5), and we assume that the collection and measurement dates were generally close. Considering the small annual rate of change (< 20%) and the length of the sampling period, it is unlikely that a substantial bias affected the conclusions.

It has been pointed out that non-destructive testing machines cannot fill samples homogeneously and can sometimes introduce a bias of > 20% vs. HPGe measurements^52^. However, in the case of wild mushrooms and plants, the variation among species and samples is substantial, on the one to two orders of magnitude. Therefore, we consider the influence of errors because of the measurement equipment to be dwarfed by other factors.

## Supplementary Information

Supplementary Information 1.

Supplementary Information 2.

Supplementary Information 3.
