# Supplementary material for: Effects of species and geo-information on the 137Cs concentrations in edible wild mushrooms and plants collected by residents after the Fukushima nuclear accident
Source: Sci Rep. 2021 Nov 17;11:22470. doi: 10.1038/s41598-021-01816-z (PMC8599460; doi:10.1038/s41598-021-01816-z)
Supplement: Supplementary file 1 — Supplementary Information 1. [file 41598_2021_1816_MOESM1_ESM.docx]

**Effect of species and geo-information on the ^137^Cs concentrations in edible wild mushrooms and plants collected by residents after the Fukushima nuclear accident**

Masabumi Komatsu, Shoji Hashimoto, Toshiya Matsuura

**
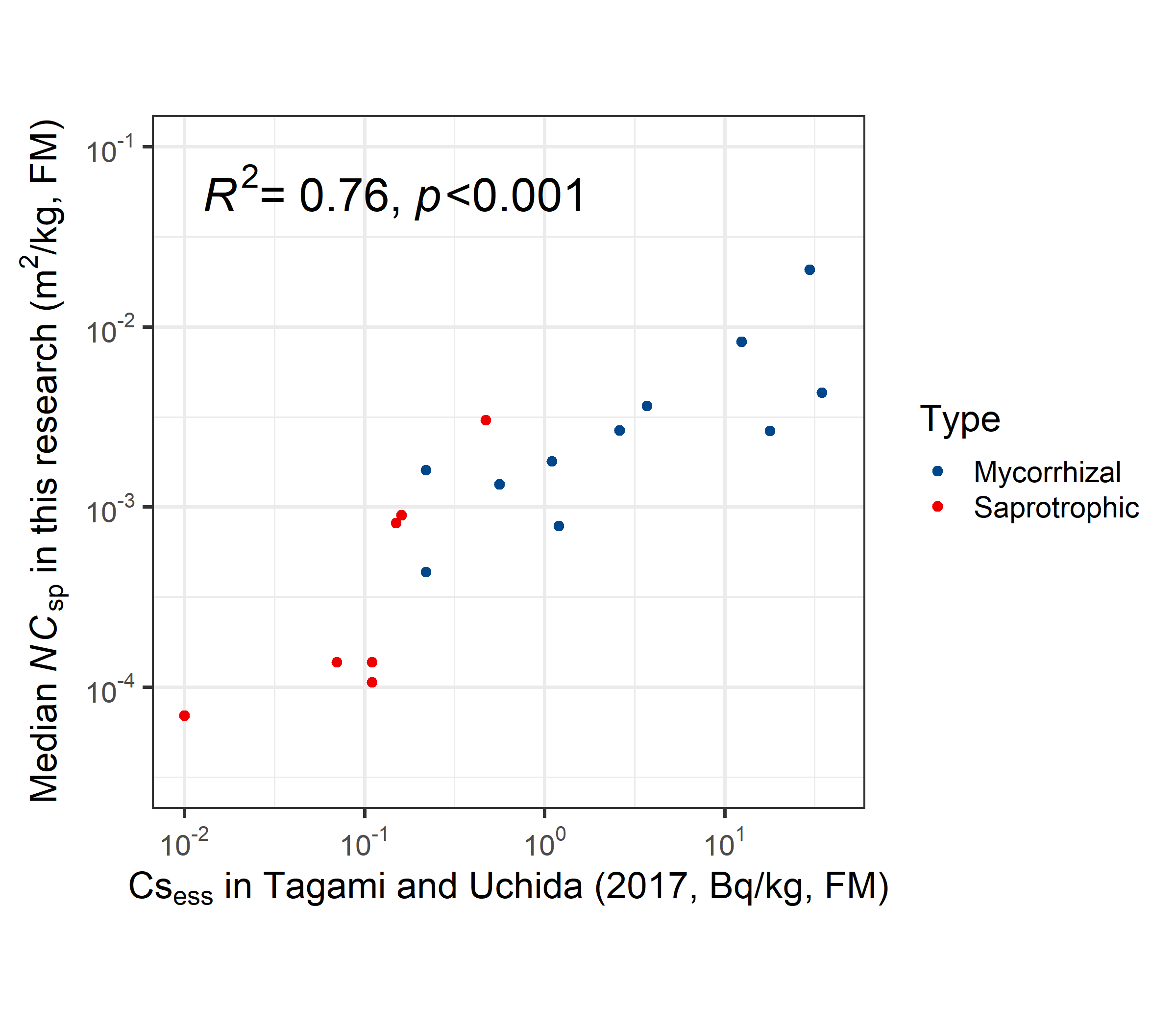
**

**Figure S1. Comparison of normalized concentration parameters of species (*NC*_sp_) in this study vs. ^137^Cs of global fallout origin (Cs_ess_) in Tagami and Uchida (2017)^31^. Only mushroom species common to both studies are considered. Weight units of both parameters are expressed as kg fresh matter (FM). The scales of both axes are logarithmic.**


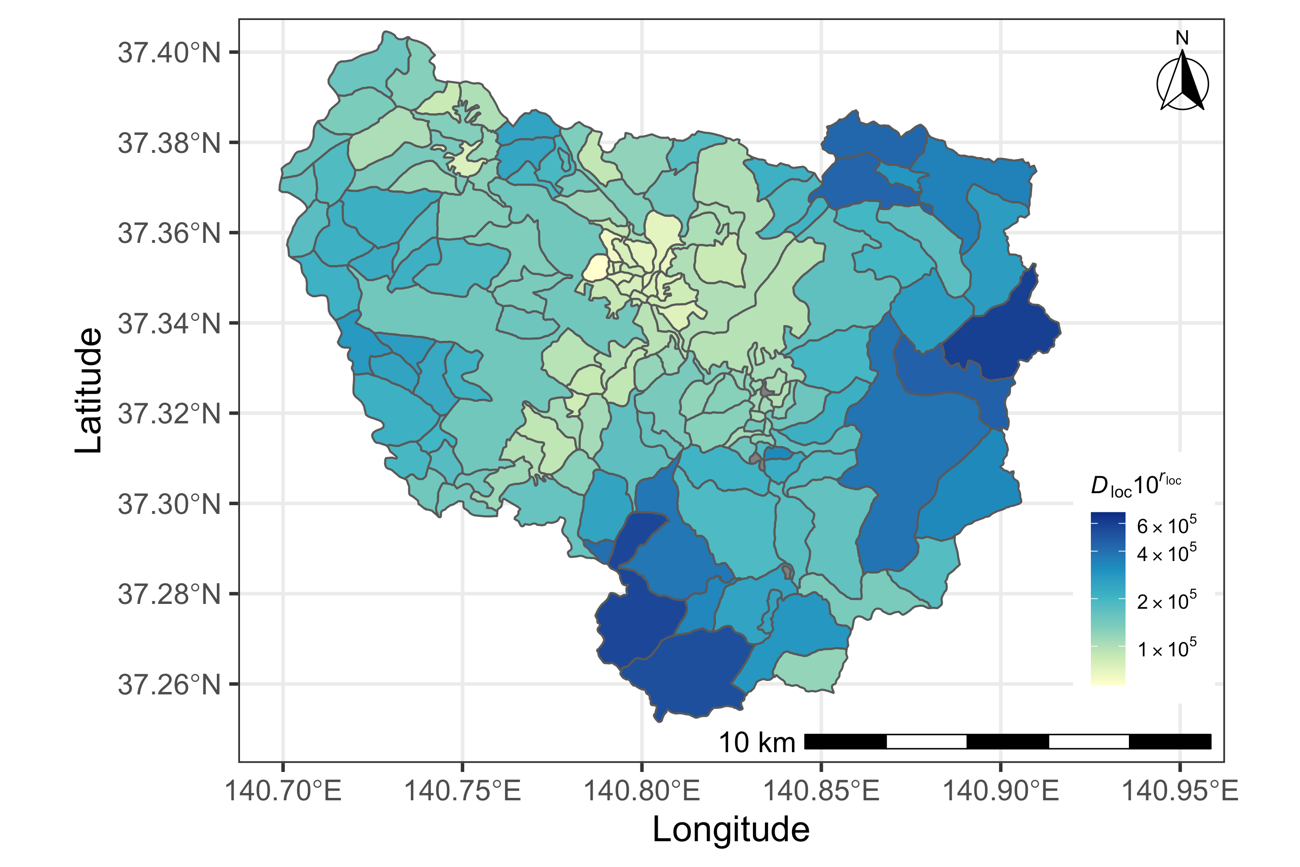


**Figure S2. The sub-village map of the product of ^137^Cs deposition (*D*_loc_) and location factor to the 10th power (10*^r^*^loc^). The sub-village boundaries were created from a hand-drawn map obtained from the administrative office. The map was created using R version 4.1.0^29^ and ggplot2 package^30^**


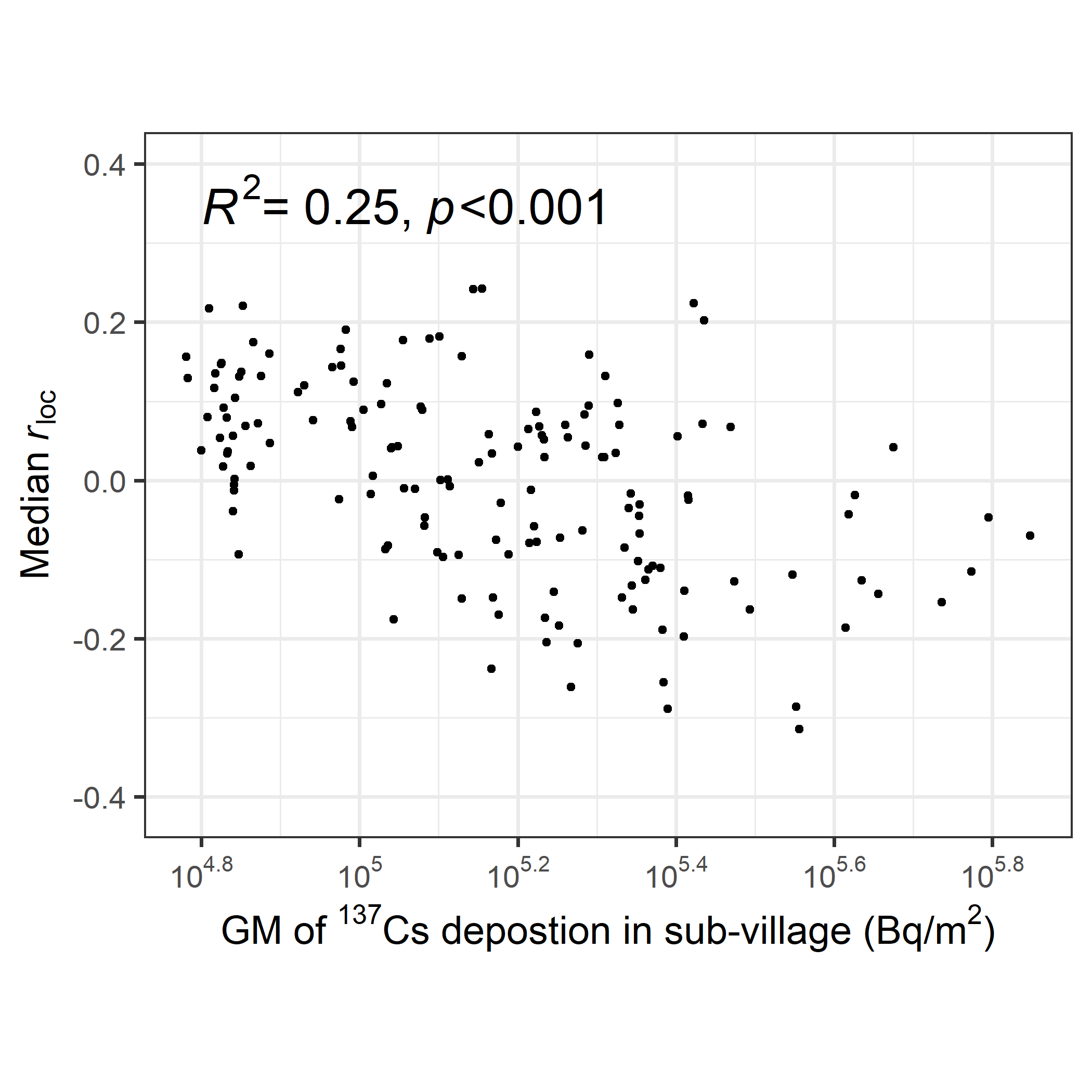


**Figure S3. Relationship between ^137^Cs deposition (*D*_loc_, geometric mean) and median *r*_loc_ of each sub-village. In this analysis, we have excluded data from Matsutake (*Tricholoma matsutake*) samples.**

**
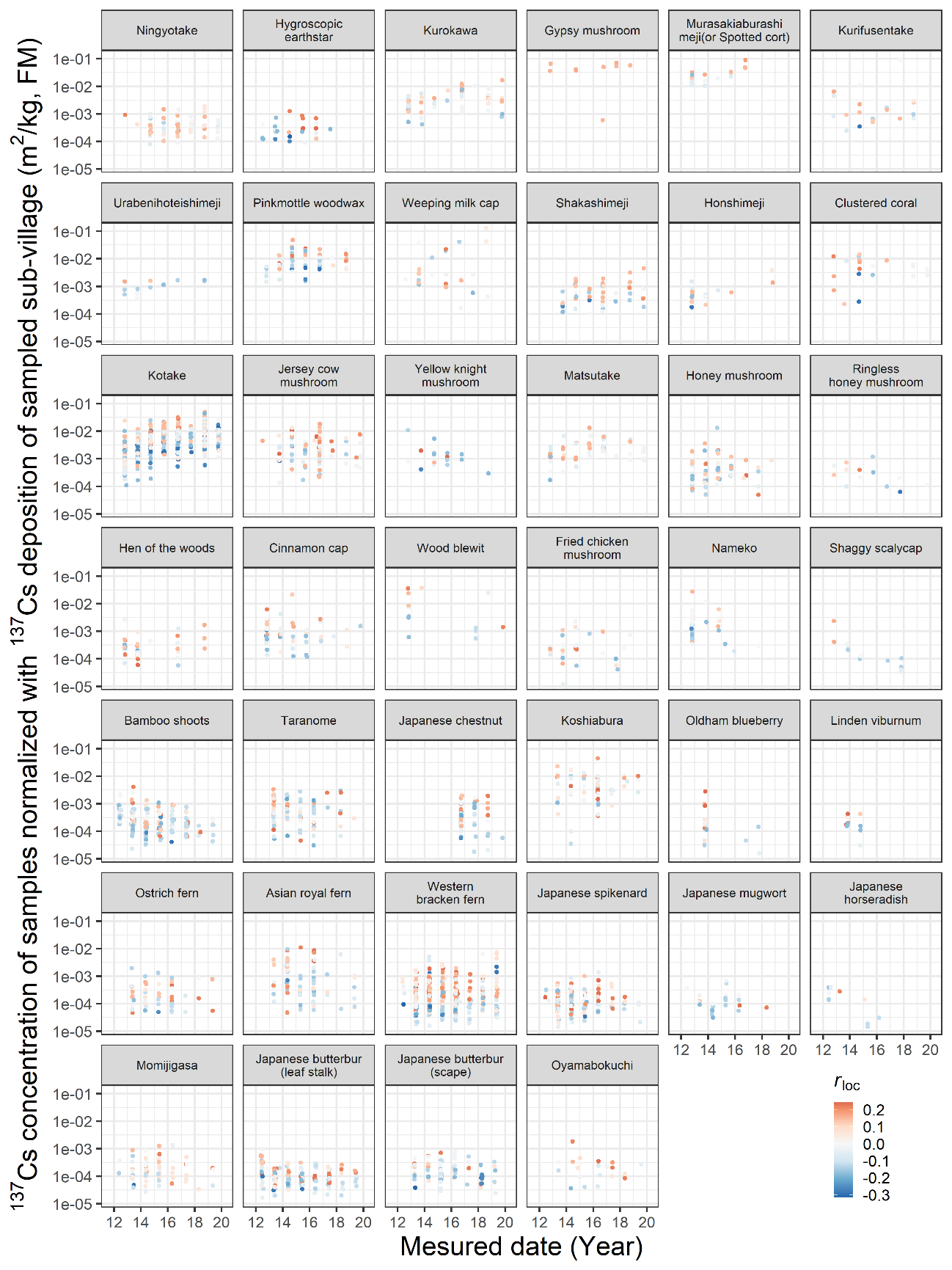
 Figure S4. The temporal trends of normalized ^137^Cs concentration of each species. Each sample (point) was indicated with the magnitude of the effect of sampled sub-village on ^137^Cs activity concentration (*r*_loc_). Only samples of which radiocesium concentration were detected, were plotted.**

**
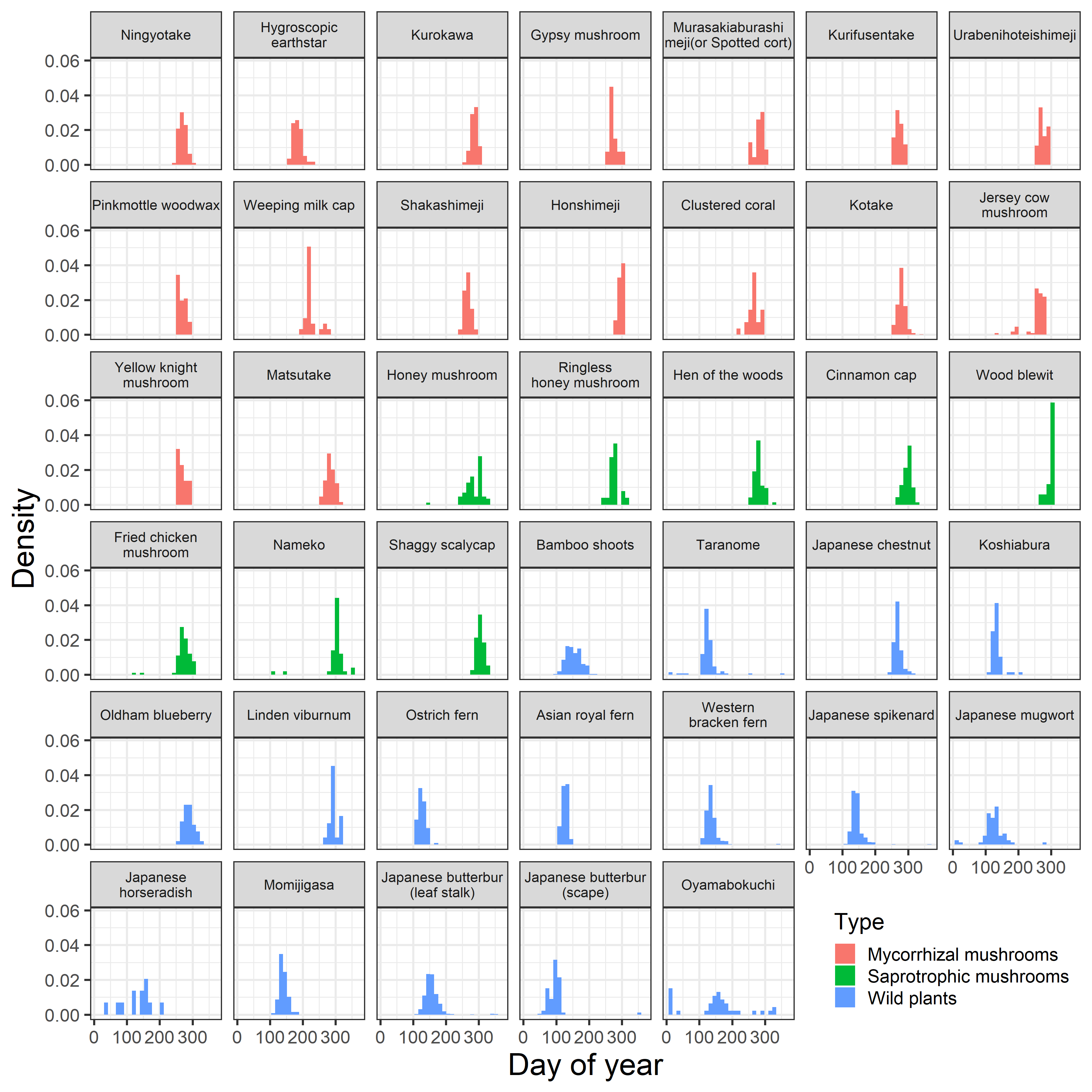
**

**Figure S5. Histograms of sampling date (day of year) according to species.**
