# Supplementary material for: Effects of species and geo-information on the 137Cs concentrations in edible wild mushrooms and plants collected by residents after the Fukushima nuclear accident
Source: Sci Rep. 2021 Nov 17;11:22470. doi: 10.1038/s41598-021-01816-z (PMC8599460; doi:10.1038/s41598-021-01816-z)
Supplement: Supplementary file 3 — Supplementary Information 3. [file 41598_2021_1816_MOESM3_ESM.docx]

# Additional information

All plant and mushroom samples involved in this study were collected by residents of Kawauchi Village for inspection. We do not own the samples used in this study because the collectors have ownership of these samples. We received the measurement radioactivity data of these samples from the administrative office of Kawauchi Village.
